# Racial and ethnic disparities in receipt of guideline-concordant treatment for early-stage non-small cell lung cancer

**DOI:** 10.1007/s10552-026-02206-4

**Published:** 2026-07-01

**Authors:** Miriam L. Gorbatov, Angel Arizpe, Elizabeth A. David, Lihua Liu, Myles Cockburn, Albert J. Farias

**Affiliations:** 1 Department of Population and Public Health Sciences, Keck School of Medicine, University of Southern California, 1845 N. Soto St., Suite 318B, Los Angeles, CA 90032 USA; 2 University of Colorado School of Medicine, Anschutz Medical Campus, Aurora, CO USA

**Keywords:** Non-small cell lung cancer, Guideline-concordant treatment, Health disparities

## Abstract

**Purpose:**

Non-small cell lung cancer (NSCLC) is the leading cause of cancer mortality in California, with Non-Hispanic Black (NHB) patients experiencing the highest mortality rates. Disparities in receipt of guideline-concordant treatment (GCT) contribute to these outcomes. This population-based study analyzed racial and ethnic differences in GCT receipt among early-stage NSCLC patients.

**Methods:**

Patients diagnosed with early-stage NSCLC (tumors ≤ 4 cm, no lymph node involvement, and no distant metastases) between 2012 and 2022 were identified from the Los Angeles County Cancer Surveillance Program. Logistic regression evaluated associations between race/ethnicity and receipt of GCT (surgery or radiation only vs. no treatment or multiple treatments), adjusting for potential confounders.

**Results:**

In our sample (*n* = 2,857), the average age was 69.6 years, 63.2% were female, mean tumor size was 1.9 cm, and the cohort was 57.3% Non-Hispanic White (NHW), 8.9% NHB, 14.2% Hispanic, and 18.8% Asian/Pacific Islander (API). While most patients (87.4%) received GCT, NHB and Hispanic patients had higher rates of non-GCT treatment (18.0% and 15.8%, respectively; *p* < 0.001). NHB patients had significantly higher odds of not receiving GCT compared with NHW patients (OR: 1.55, 95% CI: 1.07–2.26; *p* < 0.05). No significant differences were observed for API or Hispanic patients.

**Conclusion:**

Addressing disparities in GCT for patients diagnosed with early-stage NSCLC is critical to improving survival outcomes. Future studies identifying factors influencing a patient’s and physician’s decision to receive, recommend, and adhere to GCT guidelines are needed to understand the complexity of treatment decision-making.

## Background

Non-small cell lung cancer (NSCLC) is the leading cause of cancer-related mortality in the United States, and the second most commonly diagnosed cancer in California [1]. Mortality rates for NSCLC vary widely in California. While the mortality rate for early-stage NSCLC is 29 per 100,000 across all patients, the rate is lower for Hispanic (17 per 100,000) and Asian/Pacific Islanders (23 per 100,000) and higher for Black patients (39 per 100,000) compared to Non-Hispanic White patients (34 per 100,000) [1]. Potential contributors to these disparities include patient factors such as comorbidities, income, age, rural environments, regional variation in treatment, and patient-physician experiences [2–6]. Mortality rates also differ substantially by stage of diagnosis. Across the US, patients diagnosed with early-stage NSCLC have a 65% likelihood of 5-year survival compared with just 9% for patients diagnosed at a later-stage [7]. Of the 180,000 patients diagnosed with NSCLC each year, approximately one-third are diagnosed with early-stage disease and are potential candidates for curative-intent surgical resection or radiation [8–10]. For patients diagnosed with early-stage NSCLC, disparities in mortality can be reduced by improving receipt of guideline-concordant treatment (GCT), as prior studies have observed that non-white patients and patients from lower socioeconomic status groups are less likely to undergo therapeutic resection or radiation treatment [11–13]. Racial and socioeconomic disparities in stage at diagnosis also contribute to survival differences, with Black and low-income patients more often diagnosed at later stages when curative treatment options are more limited [14, 15]. The American College of Chest Physicians and the National Comprehensive Cancer Network recommend surgical resection for all medically operable patients with stage I–II NSCLC, while radiation alone is recommended for patients deemed medically inoperable [13, 16]. The role of surgery as the principal treatment for localized NSCLC is well established, and prior studies demonstrated prolonged overall survival following surgery compared to treatment with radiation therapy alone or no therapy [12, 13, 17]. However, despite rigorous clinical and oncologic evidence for these guidelines, a significantly greater proportion of Black (24.7%) and Hispanic (26.5%) Californians do not receive treatment compared to NHW patients (22.3%) [18]. This persistent underutilization of evidence-based treatment among racial and ethnic minorities raises concerns about systemic, provider, and patient-level contributors to disparities in early-stage NSCLC care. A seminal study by Bach et al. found that Black patients with early-stage NSCLC had significantly lower five-year overall survival compared to White patients, a difference largely explained by lower rates of surgical resection among Black patients [11]. Additionally, Black patients have also been found to be more likely to receive external beam radiation therapy and less likely to receive stereotactic body radiotherapy, even though the latter is considered more effective for early-stage NSCLC [19–21]. While disparities in surgical and radiation treatment for Black patients have been documented in the literature, few studies have evaluated whether Hispanic and Asian patients experience similar disparities in care. There is also a need for robust, population-level data that reflects the diverse racial and ethnic makeup of major metropolitan areas such as Los Angeles County, where longstanding disparities in lung cancer mortality persist. Our study is a population-based analysis of patients diagnosed with early-stage NSCLC using data from the Los Angeles County Cancer Surveillance Program. Differences in receipt of GCT, defined as surgery or radiation therapy, were evaluated across racial and ethnic groups, adjusting for key covariates including patient demographics, socioeconomic factors, and tumor characteristics.

## Methods

### Study design and data source

We conducted a population-based observational study using data from the Los Angeles County Cancer Surveillance Program (LAC-CSP), a SEER-affiliated cancer registry. The LAC-CSP has served as the population-based cancer registry for LAC since 1972 and systematically collects information on all incident cancer diagnoses among county residents. The CSP is a member of the statewide population-based cancer surveillance program (California Cancer Registry) and contributes data to the National Cancer Institute’s Surveillance, Epidemiology, and End Results SEER program. LAC is the most populous county in the United States, with over 10 million residents. According to 2020 U.S. Census data, the racial and ethnic composition of the county is 48% Hispanic or Latino ethnicity, 32.6% White, 8.0% Black, 1.6% American Indian/Alaskan Native, 15% Asian, and 0.2% Native Hawaiian/Pacific Islander [22]. Our analysis included patients diagnosed with early-stage NSCLC between January 1, 2012 and December 31, 2022.

### Cohort selection

A total of 2,857 patients were identified from the 2012 to 2022 LAC-CSP registry files. These patients met the inclusion criteria for the clinical definition of early-stage NSCLC defined as tumor size ≤ 4 cm, no regional lymph node involvement, and no evidence of distant metastases.

### Statistical analysis plan

Descriptive statistics (including age, sex, marital status, insurance type, tumor size, stage at diagnosis, and socioeconomic status) were summarized across racial and ethnic groups and by treatment type. Means and standard deviations are reported for continuous variables and proportions were calculated for categorical variables. To compare baseline demographic and clinical characteristics by treatment type, we conducted bivariate analyses using chi-square tests for categorical variables and analysis of variance for continuous variables.

The primary outcome was receipt of GCT, defined as surgical resection or radiation only, consistent with guidelines for medically operable patients with early-stage NSCLC from the National Comprehensive Cancer Network and American College of Chest Physicians. Radiation modality was not available in the registry data, so any recorded radiation treatment was classified as guideline-concordant radiation. The primary exposure was race and ethnicity categorized as Non-Hispanic White (NHW), Non-Hispanic Black (NHB), Hispanic, Asian/Pacific Islander (API), or Other. We used logistic regression to estimate the crude association between race/ethnicity and the likelihood of receiving GCT. We fit a series of nested models to sequentially adjust for potential confounders including age, sex, marital status, tumor size, insurance type, and SES. This sequential modeling approach provided insight into the individual and combined effects of each covariate on observed treatment disparities.

Odds ratios and 95% confidence intervals were reported for each model. Insurance type was categorized as private, Medicaid, Medicare, or uninsured/other. Socioeconomic status (SES) was assessed using a census tract-level SES quintile provided by the California Cancer Registry. For cases diagnosed after 2006, SES in the CCR is measured using Juan Yang’s index of socioeconomic status with missing values imputed based on principal component analysis of block group-level variables from the American Community Survey [23]. Effect measure modification was evaluated by including interaction terms between race/ethnicity and age, sex, insurance type, and SES. All analyses were conducted using SAS 9.4 (SAS Institute, Cary, NC), and statistical significance was determined at a *p* < 0.05 (RRID: SCR_008567). Individual patient consent was not required as this study used cancer registry data collected for public health surveillance and analyzed under approved data use agreements in accordance with institutional and state guidelines.

## Results

Among 2,857 patients diagnosed with early-stage NSCLC, 57.3% were NHW, 8.9% were NHB, 14.2% were Hispanic, and 18.8% were API. The mean age at diagnosis was 69.6 years, with NHW patients slightly older on average (70.7 years). Females made up 63.2% of the sample. Marital status and socioeconomic status varied by race/ethnicity. A greater proportion of NHB patients were single (29.4%) compared with NHW patients (15.5%), while API patients were most likely to be married (66.7%). NHB and Hispanic patients were overrepresented in the lowest SES quintile (27.5 and 26.4%, respectively), compared with NWH patients (4.6%). Tumor size was also slightly larger among Hispanic patients (mean = 20.2 mm) compared with other racial and ethnic groups (Table 1).

**Table 1:** Baseline characteristics of early-stage NSCLC by race and ethnicity in Los Angeles County (2012–2022) (*n* = 2857)

Characteristic	Non-Hispanic White	Non-Hispanic Black	Hispanic	Asian/Pacific Islander	Other	Total
Age						
Mean (SD)	70.69 (9.15)	67.78 (9.82)	67.55 (11.71)	68.93 (10.02)	67.83 (8.61)	69.63 (9.85)
Sex, *n* (%)						
Male	632 (38.68)	94 (36.86)	131 (32.35)	187 (34.82)	8 (33.33)	1052 (36.85)
Marital status, *n* (%)						
Single, never married	253 (15.46)	75 (29.41)	89 (21.98)	52 (9.68)	1 (4.17)	472 (16.45)
Married	873 (53.36)	96 (37.65)	205 (50.62)	358 (66.67)	13 (54.17)	1545 (54.08)
Other	510 (31.17)	84 (32.94)	111 (27.41)	127 (23.65)	10 (41.67)	842 (29.47)
Insurance type, *n* (%)						
Private	504 (31.38)	80 (31.87)	129 (32.74)	182 (35.27)	11 (45.83)	906 (32.46)
Medicaid	44 (2.69)	21 (8.24)	62 (15.74)	39 (7.56)	2 (8.33)	168 (5.88)
Medicare	1049 (65.32)	150 (59.76)	201 (51.02)	292 (56.59)	9 (37.50)	1701 (60.95)
Uninsured/other/unknown	24 (4.47)	13 (3.21)	4 (1.57)	39 (2.38)	2 (8.33)	82 (2.87)
Tumor size, mm						
Mean (SD)	18.57 (8.36)	18.75 (8.54)	20.17 (8.77)	19.05 (8.41)	17.68 (7.52)	18.90 (8.45)
Stage at diagnosis, *n* (%)						
IA	1273 (77.81)	198 (77.65)	304 (75.06)	411 (76.54)	19 (79.17)	2205 (77.18)
IB	363 (22.19)	57 (22.35)	101 (24.94)	126 (23.46)	5 (20.83)	652 (22.82)
Treatment type, *n* (%)						
Guideline-concordant surgery	1269 (77.57)	181 (70.98)	306 (75.56)	427 (79.52)	16 (66.67)	2199 (76.97)
Guideline-concordant radiation	190 (11.61)	28 (10.98)	35 (8.64)	42 (7.82)	2 (8.33)	297 (10.4)
Guideline-discordant treatment	91 (5.56)	22 (8.63)	43 (10.62)	40 (7.45)	3 (12.5)	199 (6.97)
No treatment/unknown	86 (5.26)	24 (9.41)	21 (5.19)	28 (5.21)	3 (12.5)	162 (5.67)
SES quintile						
Lowest SES	75 (4.58)	70 (27.45)	107 (26.42)	52 (9.68)	1 (4.17)	305 (10.68)
Lower-middle SES	170 (10.39)	49 (19.22)	115 (28.40)	117 (21.79)	4 (16.67)	455 (15.93)
Middle SES	319 (19.50)	66 (25.88)	85 (20.99)	112 (20.86)	6 (25.00)	588 (20.58)
Upper-middle SES	467 (28.55)	49 (19.22)	61 (15.06)	133 (24.77)	8 (33.33)	718 (25.13)
Highest SES	605 (36.98)	21 (8.24)	37 (9.14)	123 (22.91)	5 (20.83)	791 (27.69)

Across all racial and ethnic groups, most patients received guideline-concordant surgery (*n* = 2199; 77%), while 10.4% received guideline-concordant radiation, 7.0% received discordant treatment, and 5.7% received no treatment. Treatment patterns varied by race and ethnicity. NHB patients had lower rates of guideline-concordant surgery than NHW patients (71.0 vs. 77.6%) and higher rates of non-GCT (18.0 vs. 10.8%). The proportion receiving non-GCT was 15.8% among Hispanic patients and 12.7% among API patients. Marital status, insurance type, tumor size, stage at diagnosis, and SES were all significantly associated with treatment receipt. Patients in the highest SES quintile were more likely to receive guideline-concordant surgery compared with those in the lowest quintile (Table 2).

**Table 2:** Baseline characteristics of early-stage NSCLC by treatment type in Los Angeles County (2012–2022) (*n* = 2,857)

Characteristic	Guideline concordant radiation	Guideline concordant surgery	Guideline discordant treatment	No treatment/unknown	Total	*p*-value
Age						
Mean (SD)	75.70 (8.29)	68.72 (9.75)	68.79 (8.81)	71.37 (10.53)	69.63 (9.85)	<0.001
Sex, *n* (%)						
Male	130 (43.77)	798 (36.31)	59 (36.42)	66 (33.17)	1053 (36.86)	0.05
Race/ethnicity, *n* (%)						
White	190 (63.97)	1269 (57.71)	86 (53.09)	91 (45.73)	1636 (57.26)	0.001
Black or African American	28 (9.43)	181 (8.23)	24 (14.81)	22 (11.06)	255 (8.93)	
Asian	42 (14.14)	427 (19.42)	28 (17.28)	40 (20.10)	537 (18.80)	
Hispanic	35 (11.78)	306 (13.92)	21 (12.96)	43 (21.61)	405 (14.18)	
Other	2 (0.67)	16 (0.73)	3 (1.85)	3 (1.51)	24 (0.84)	
Marital status, *n* (%)						
Single, never married	51 (17.17)	350 (15.92)	21 (12.96)	48 (24.12)	470 (16.45)	<0.001
Married	141 (47.47)	1222 (55.57)	97 (59.88)	85 (42.71)	1545 (54.08)	
Other	105 (35.35)	627 (28.51)	44 (27.16)	66 (33.17)	842 (29.47)	
Insurance type, *n* (%)						
Private	58 (19.53)	753 (34.24)	54 (33.33)	41 (20.60)	906 (31.71)	<0.001
Medicaid	14 (4.71)	124 (5.64)	5 (3.09)	25 (12.56)	166 (5.88)	
Medicare	217 (73.06)	1260 (57.30)	97 (59.88)	127 (63.82)	1701 (59.54)	
Other/unknown	8 (2.69)	62 (2.82)	6 (3.70)	6 (3.02)	82 (2.87)	
Tumor size, mm						
Mean (SD)	19.07 (8.26)	18.68 (8.17)	23.31 (8.26)	19.61 (8.49)	19.04 (8.27)	<0.001
Stage at diagnosis, *n* (%)						
IA	260 (87.54)	1698 (77.22)	76 (46.91)	171 (85.93)	2205 (77.18)	<0.001
IB	37 (12.46)	501 (22.78)	86 (53.09)	28 (14.07)	652 (22.82)	
SES quintile						
Lowest SES	27 (9.09)	227 (10.32)	25 (15.43)	26 (13.07)	305 (10.68)	0.011
Lower-middle SES	51 (17.17)	336 (15.28)	27 (16.67)	41 (20.60)	455 (15.93)	
Middle SES	67 (22.56)	436 (19.83)	34 (20.99)	51 (25.63)	588 (20.58)	
Upper-middle SES	69 (23.23)	560 (25.47)	44 (27.16)	45 (22.61)	718 (25.13)	
Highest SES	83 (27.95)	640 (29.10)	32 (19.75)	26 (18.09)	791 (27.69)	

In the unadjusted model, NHB patients had significantly higher odds of not receiving GCT compared with NHW patients (OR = 1.81; 95% CI: 1.27–2.59, *p* < 0.01) (Table 3). Hispanic patients also had 55% higher odds of non-receipt of GCT compared with NHW patients (OR = 1.55; 95% CI: 1.14–2.11, *p* < 0.01), whereas differences for API patients were not statistically significant. After adjusting for all covariates, the odds of receiving non-GCT remained significantly higher for NHB patients (OR = 1.55; 95% CI: 1.07–2.26, *p* < 0.05), but the association for Hispanic patients was attenuated and no longer significant (OR = 1.23; 95% CI: 0.88–1.73).

**Table 3:** Association between race/ethnicity and non-receipt of guideline-concordant treatment in crude and adjusted models

	Model 1: Crude (*n* = 2857)	Model 6: Adjusted (*n* = 2857)
	OR (95% CI)	OR (95% CI)
Race and ethnicity		
Non-Hispanic Black vs. NHW	1.81 (1.27–2.59)**	1.55 (1.07–2.26)*
Hispanic vs. NHW Asian/Pacific Islander vs. NHW Other vs. NHW	1.55 (1.14–2.11)** 1.20 (0.89–1.61) 2.75 (1.08–7.01) *	1.23 (0.88–1.73) 1.09 (0.80–1.49) 2.70 (1.04–7.02)*
Age	–	1.01 (1.00–1.02)
Female vs. male	–	1.13 (0.89–1.44)
Married vs. single	–	0.89 (0.65–1.22)
Other marital status vs. single	–	0.86 (0.61–1.21)
Insurance-medicaid	–	1.49 (0.93–2.38)
Insurance-medicare	–	1.26 (0.95–1.67)
Insurance-not insured/other/unknown	–	1.44 (0.74–2.77)
Tumor size	–	1.04 (1.02–1.05)
SES quintile lower-middle SES	–	1.04 (0.60–1.35)
SES quintile middle SES	–	0.90 (0.60–1.35)
SES quintile upper-middle SES	–	0.93 (0.63–1.39)
SES quintile highest SES	–	0.84 (0.56–1.25)
		0.56 (0.37–0.86)

Fully adjusted model is adjusted for age, sex, marital status, tumor size, SES quintiles, and insurance. For SES, the lowest quintile serves as the reference group

*NHW,* Non-Hispanic White

*p < 0.05, **p < 0.01

In the multivariate regression models, after adjusting for age and sex, NHB and Hispanic patients had higher odds of non-receipt of GCT compared with NHW patients (Table 4). However, the estimates (while still significant in models 3 and 4) were attenuated for both NHB and Hispanic patients after including marital status, tumor size, and insurance into the regression model (Table 4). For Hispanic patients, the disparities observed in the crude model and then in models 2–4 (adjusting for age, sex, marital status, tumor size, and insurance) are largely explained by differences in socioeconomic status (Table 4). In Model 5, after adjusting for SES, there was no significant difference in non-receipt of GCT between Hispanic and NHW patients (OR = 1.26, 95% CI: 0.90–1.76). However, among NHB patients, even after adjusting for SES, patients remained significantly less likely to receive treatment compared with NHW patients (OR = 1.56; 95% CI: 1.07–2.27).

**Table 4:** Association between race/ethnicity and non-receipt of guideline-concordant treatment with incremental adjustment for confounders

	Model 2: Adjusted (age and sex) (*n* = 2857)	Model 3: Adjusted (age, sex, marital status, tumor size) (*n* = 2857)	Model 4: Adjusted (age, sex, marital status, tumor size, insurance) (*n* = 2857)	Model 5: Adjusted (age, sex, marital status, tumor size, SES) (*n* = 2857)
	OR (95% CI)	OR (95% CI)	OR (95% CI)	OR (95% CI)
Race and ethnicity				
Non-Hispanic Black vs. NHW	1.87 (1.31–2.67)**	1.81 (1.26–2.60)**	1.80 (1.25–2.58)**	1.56 (1.07–2.27)*
Hispanic vs. NHW	1.59 (1.16–2.17)**	1.47 (1.08–2.02)*	1.43 (1.03–1.97)*	1.26 (0.90–1.76)
Asian/pacific islander vs. NHW	1.21 (0.90–1.64)	1.20 (0.89–1.63)	1.18 (0.87–1.60)	1.11 (0.81–1.51)
Other vs. NHW	2.82 (1.10–7.21)*	2.86 (1.11–7.37)*	2.86 (1.10–7.40)*	2.69 (1.04–6.96)*

*NHW,* Non-Hispanic White

*p < 0.05, **p < 0.01

## Discussion

In this population-based study of patients diagnosed with early-stage NSCLC in Los Angeles County, we observed persistent racial and ethnic disparities in the receipt of GCT. Los Angeles County is highly diverse, with substantial racial, ethnic, and socioeconomic variation, making it an important setting for examining disparities in cancer care access and outcomes.

Our findings indicate that the majority of patients received clinically recommended guideline-concordant care (surgery or radiation). However, some patients, particularly Non-Hispanic Black patients, had significantly higher odds of not receiving GCT compared with Non-Hispanic White patients, after adjusting for sociodemographic, clinical, and insurance-related factors. While this disparity is concerning, it aligns with several national studies that have observed similar results. Analyses across other cohorts have continued to demonstrate persistent disparities in receipt of surgical resection among Black patients, despite the extensive clinical guidelines intended to standardize care regimens [24–26]. These disparities are not unique to NSCLC, with similar patterns observed across other cancers diagnosed at an early stage such as breast, pancreatic, and esophageal cancer [27–29]. This persistent disparity was observed despite decades of clinical guidelines emphasizing the importance of surgical resection and radiation, and was observed within a large, urban setting like Los Angeles County. This suggests there may be other systemic, interpersonal, or healthcare-related barriers that contribute to undertreatment among NHB patients. These include treatment-related beliefs, cultural factors, and lack of trust within the healthcare system [30–32]. At the healthcare system level, access to care, differences in insurance or referrals for surgical consultation, or miscommunication may be driving disparities in surgical care for Black patients with early-stage NSCLC.

For Hispanic patients, higher odds of receiving non-GCT were observed in the crude model as well as models adjusting for age, sex, marital status, and insurance. Although the effect estimate was attenuated with the addition of more covariates, the association remained statistically significant until socioeconomic status (SES) was included in the model. This may suggest that economic barriers and perhaps even neighborhood-level conditions may contribute to treatment disparities among Hispanic patients. However, other unmeasured factors including comorbidities, smoking history, functional status, patient preferences, and treatment eligibility may also influence receipt of GCT. This is in contrast with Non-Hispanic Black patients, where disparities in treatment persisted even after adjusting for SES, indicating that interpersonal and healthcare system barriers play a more prominent role in driving treatment differences. For Asian/Pacific Islander patients, the crude and all iteratively adjusted models showed no significant difference in GCT compared to Non-Hispanic White patients. Interaction terms for race and ethnicity with age, insurance status, and SES did not reach statistical significance for effect measure modification.

In addition to timely receipt of GCT, routine screening for high-risk patients is also critical to reducing lung cancer mortality. Current guidelines from the US Preventive Services Task Force recommend low-dose CT screening for individuals aged 50–80 years with a 20-pack-year smoking history who currently smoke or have quit within the past 15 years. However, the prevalence of lung cancer screening is universally low (16.4% in 2022) across the US [33]. Disparities in screening uptake are especially pronounced among Black, Hispanic, and low-income populations which contributes to later-stage diagnoses and limited treatment options [34, 35]. Improving screening across populations will lead to more early-stage NSCLC diagnoses and therefore, identifying GCT treatment pathways will be important to reducing racial/ethnic mortality disparities.

This study serves as the foundation for our future work in understanding the multilevel factors driving treatment disparities in early-stage NSCLC in Los Angeles County. While our findings confirm that significant disparities persist, particularly among Non-Hispanic Black patients, they also highlight the variation in factors driving guideline-concordant care. Future work will use a mixed-methods study design to integrate registry data with primary survey data from physicians and their treated NSCLC patients to examine physician-level factors such as referral patterns, guideline adherence, and barriers to providing GCT and patient-level factors such as treatment beliefs, support systems, and financial restrictions. This will provide information on the main factors influencing physician care recommendations and patient decision-making and lead to an improved understanding of the persistent disparities in guideline-concordant care for patients diagnosed with early-stage NSCLC. Additionally, as lung cancer screening uptake improves over time, more patients will be diagnosed at earlier stages. When combined with equitable access to GCT, this has the potential to significantly reduce racial and ethnic disparities in lung cancer mortality.

There are several limitations in this study. Although the Los Angeles County Cancer Surveillance Program provides detailed, population-based registry data, information influencing treatment decisions, such as patient preferences, cultural beliefs, and health literacy, was unable to be accounted for in the analysis. The registry data also contained limited information on patient comorbidities, which are important determinants of eligibility for surgery or radiation. Additionally, the registry data limited our ability to distinguish stereotactic body radiation therapy from conventional external beam radiation therapy; therefore, we were unable to evaluate differences in the receipt of specific radiation modalities.

Our findings confirm significant differences in patient receipt of GCT, particularly for Non-Hispanic Black patients. While economic barriers may drive disparities among Hispanic patients, systemic, interpersonal, or patient health-related factors may play a more prominent role in treatment disparities for Non-Hispanic Black patients. As lung cancer screening rates improve and more patients are diagnosed at earlier stages, ensuring equitable access to and uptake of recommended treatment will be critical for reducing racial and ethnic disparities in lung cancer outcomes.

## Data Availability

The data used in this study was obtained from the Los Angeles County Cancer Surveillance Program (LAC-CSP), a SEER-affiliated, population-based cancer registry. Access to LAC-CSP is restricted and subject to approval by the University of Southern California’s Cancer Surveillance Program and other regulatory bodies to ensure compliance with data use agreements and patient confidentiality regulations. Researchers interested in accessing LAC-CSP must submit a formal data request through LAC-CSP.
